# Homozygous carriers of the *TCF7L2* rs7903146 T-allele show altered postprandial response in triglycerides and triglyceride-rich lipoproteins

**DOI:** 10.1038/srep43128

**Published:** 2017-02-21

**Authors:** L. Engelbrechtsen, T. H. Hansen, Y. Mahendran, P. Pyl, E. Andersson, A. Jonsson, A. Gjesing, A. Linneberg, T. Jørgensen, T. Hansen, H. Vestergaard

**Affiliations:** 1 Section of Metabolic Genetics, Novo Nordisk Research Foundation Center for Basic Metabolic Research, Faculty of Health Sciences, University of Copenhagen, Universitetsparken 1, DIKU-building 1. Floor, DK-2100 Copenhagen; 2 Danish Diabetes Academy, Odense, Denmark; 3 Research Centre for Prevention and Health, The Capital Region of Denmark, Denmark; 4 Department of Clinical Experimental Research, Rigshospitalet, Denmark; 5 Department of Clinical Medicine, Faculty of Health and Medical Sciences, University of Copenhagen, Copenhagen, Denmark; 6 Department of Public health, Faculty of Health Science, University of Copenhagen, Denmark; 7 Faculty of Medicine, Aalborg University, Denmark; 8 Steno Diabetes Center, Gentofte, Denmark

## Abstract

The *TCF7L2* rs7903146 T-allele shows the strongest association with type 2 diabetes (T2D) among common gene variants. The aim of this study was to assess circulating levels of metabolites following a meal test in individuals carrying the high risk rs790346 TT genotype (cases) and low-risk CC genotype (controls). Sixty-two men were recruited based on *TCF7L2* genotype, 31 were TT carriers and 31 were age- and BMI-matched CC carriers. All participants consumed a test meal after 12 hours of fasting. Metabolites were measured using proton nuclear magnetic resonance (NMR) spectroscopy. Metabolomic profiling of *TCF7L2* carriers were performed for 141 lipid estimates. TT carriers had lower fasting levels of L-VLDL-L (total lipids in large very low density lipoproteins, p = 0.045), L-VLDL-CE (cholesterol esters in large VLDL, p = 0.03), and L-VLDL-C (total cholesterol in large VLDL, p = 0.045) compared to CC carriers. Additionally, TT carriers had lower postprandial levels of total triglycerides (TG) (q = 0.03), VLDL-TG (q = 0.05, including medium, small and extra small, q = 0.048, q = 0.0009, q = 0.04, respectively), HDL-TG (triglycerides in high density lipoproteins q = 0.037) and S-HDL-TG (q = 0.00003). In conclusion, TT carriers show altered postprandial triglyceride response, mainly influencing VLDL and HDL subclasses suggesting a genotype-mediated effect on hepatic lipid regulation.

Variants within the transcription factor 7-like 2 gene (*TCF7L2*) are the strongest genetic risk factors associated with development of type 2 diabetes (T2D)^1^. Several variants within this gene have been shown to increase the risk of diabetes by 30–50%; in which the rs7903146 has the largest and most consistent effect on diabetes susceptibility^1^.

Transcription factor 7-like 2 (TCF7L2) is involved in the Wnt signaling pathways in various tissues including muscle, fat, liver and brain cells. Carriers of *TCF7L2* risk variants have decreased insulin production, β-cell dysfunction and altered hepatic gluconeogenesis, possibly influenced by modulation of the incretin response^2^,^3^,^4^,^5^,^6^. The biological mechanism mediating these alterations is largely unknown, yet altered expression or splicing of *TCF7L2* has been suggested as a likely mechanism. Several studies have assessed the effect of the high risk T-allele on expression levels of *TCF7L2* in different tissues. Reduced expression of *TCF7L2* was reported in beta-cells from diabetic islets from T allele carriers^7^, while a marked increase in *TCF7L2* mRNA in human pancreatic islets was found^8^. However, the T allele does not seem to have an effect on the expression of *TCF7L2* in skeletal muscle and adipose tissue^9^.

Recently, Norton *et al*. demonstrated that silencing of *TCF7L2* in hepatocytes led to changes in the expression of 406 genes including key regulators of cellular growth, differentiation, and of amino acid, lipid and glucose metabolism in hepatocytes^10^, suggesting that TCF7L2 may mediate a variety of actions in hepatocytes including changes in lipid metabolism. Additionally, a meta-analysis including 24 studies with 52,575 individuals demonstrated that the minor T-allele was associated with lower triglyceride levels in individuals with T2D^11^. Thorough examination of the human metabolome following an oral glucose tolerance test in 15 high risk and 15 low risk homozygous TCF7L2 variant carriers revealed no differences among T and C allele carriers^12^, whereas an intravenous glucose tolerance test revealed changes in plasma sphingomyelins, phosphatidylcholines and lysophosphatidylcholines species in T allele carriers^13^.

Based on these findings, we aimed to perform a deep characterization of the changes that occur in lipid metabolites during a meal test in *TCF7L2* variant carriers by using a nuclear magnetic resonance spectroscopy (NMR) platform. We hypothesized that normoglycemic homozygous T-allele carriers have alterations in their lipid profile reflecting a genotype-mediated effect on lipid metabolism, which arises prior to glucose intolerance and diabetes.

## Results

Metabolite changes were observed during a standardized meal test among 31 CC carriers and 31 TT carriers. Groups were age and BMI matched and no significant differences between the groups were found in the abovementioned measures (Table 1).

**Figure 1:**
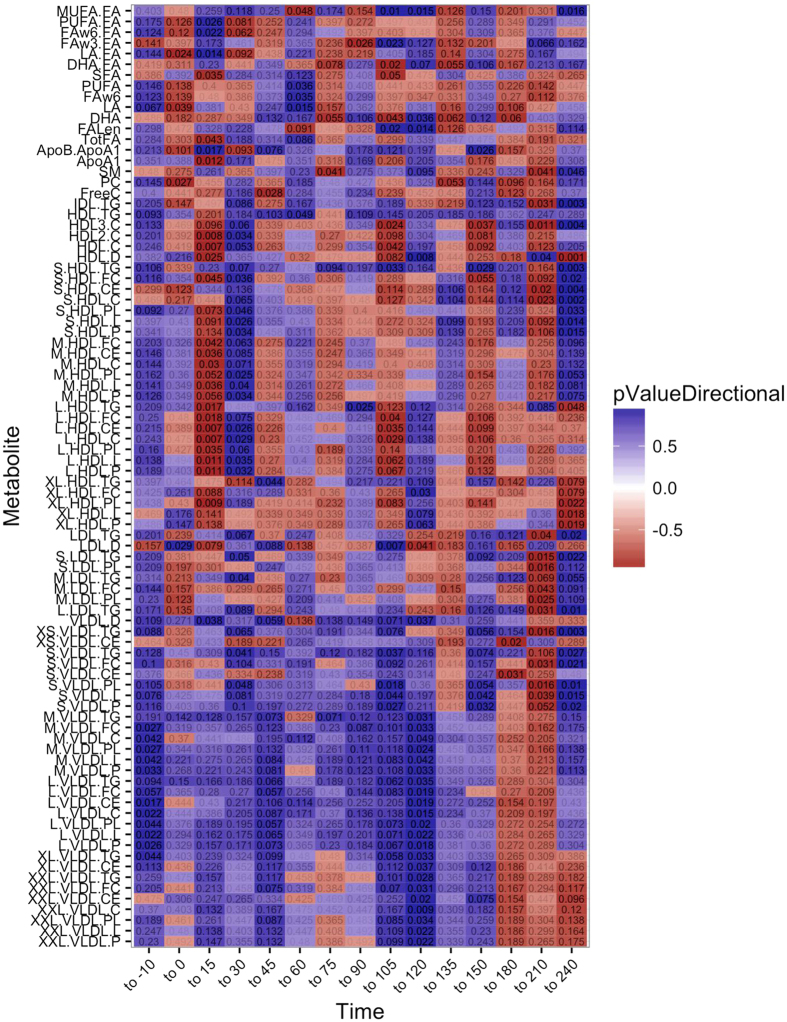
Differences in lipid response among CC and TT carriers during a meal test. A Wilcoxon Man-Whitney test was performed in each metabolite for each relative change, comparing the distributions between the two genotype of patients (CC and TT). The Heatmap visualises the resulting p-values for a selection of metabolites with significant differences (p < 0.05) at at least one of the time points. The actual p-values are overlaid as text with darker colour indicating more significant p-values. Time points reflect the difference in metabolite levels from the previous time point and compared between the groups. P-values are calculated by wilcoxon rank sum test. MUFA.FA: Ratio of monounsaturated fatty acid to total fatty acids; FAw6.FA: Ratio of omega 6 fatty acids to total fatty acids; FAw3.FA: Ratio of omega-3 fatty acids to total fatty acids; LA.FA: Ratio of 18:2 linoleic acid to total fatty acids; DHA.FA: Ratio of 22:6 docosahexaenoic acid to total fatty acids; LA: 18:2 linoleic acid; FALen: Estimated description of fatty acid chain length; ApoB.ApoA1: Ratio of ApoB and ApoA1; S.: small sixed; M.: Medium sized; L.: Large size; XL.: extra large size; XXL: Chylomicrones; VLDL: very low density lipo proteins, LDL: low density lipoproteins; IDL: Intermediate density lipoproteins; HDL: High density lipoproteins;.TG = Concentration of triglycerides in the lipoprotein;.C = Total cholesterol in the lipoprotein;.P = Total concentration of the lipoprotein;.CE = Concentration of cholesterolesters in the lipoprotein;.FC = Free cholesterol in the lipoprotein;.L: Total lipids in the lipoprotein; HDL3.C: Total cholesterol in HDL3; HDL2.C; Total cholesterol in HDL2; HDL.D: Mean diameter for HDL particles.

Metabolomic profiling of plasma samples at the 16 time points were completed for 141 lipid metabolites. Fasting levels of lipid estimates were compared using Wilcoxon rank sum test, however, only L-VLDL-L (total lipids in large VLDL, p = 0.045), L-VLDL-CE (cholesterol esters in large VLDL, p = 0.03), and L-VLDL-C (total cholesterol in large VLDL, p = 0.045) and LA:FA (ratio of 18:2 linoleic acid to total fatty acids, p = 0.048) were significantly different between the groups, with TT carriers exhibiting the lowest concentrations. Especially at the final stage of the meal test (240 min), TT carriers had lower levels of XS-VLDL-TG (triglycerides in extra small VLDLs, p = 0.006), S-HDL-TG (triglycerides in small HDLs, p = 0.006), LDL-TG (total triglycerides in LDLs, p = 0.038) and IDL-TG (total triglycerides in IDLs, p = 0.005). Overall there seemed to be a trend towards lower lipid levels in TT carriers at 240 min in many lipid fractions including S-VLDLs and S-HDLs (Fig. 1).

Mixed linear models demonstrated significant effects of the genotype on the rate of change in lipid levels during the mixed meal test. The rate of change of 39 lipid metabolites was different among groups at a FDR of 5% (Supplementary Table 1), while 9 reached Bonferroni-corrected significance (Table 2). TT carriers had lower postprandial levels of total triglycerides (q = 0.03), lower concentration of triglycerides in VLDL (q = 0.05),(medium, small and extra small sized, q = 0.048, q = 0.0009, q = 0.04, respectively)), lower concentration of triglycerides in small HDL particles (q = 0.00003), and lower ratio of monounsaturated fatty acids/total fatty acids (MUFA/FA, q = 0.006). In order to test if TCF7L2 regulate lipid levels in an insulin independent manner, we compared mixed models with and without adjustments for insulin by an anova. We found minor changes in p-values, but no changes in significance levels.

**Figure 2:**
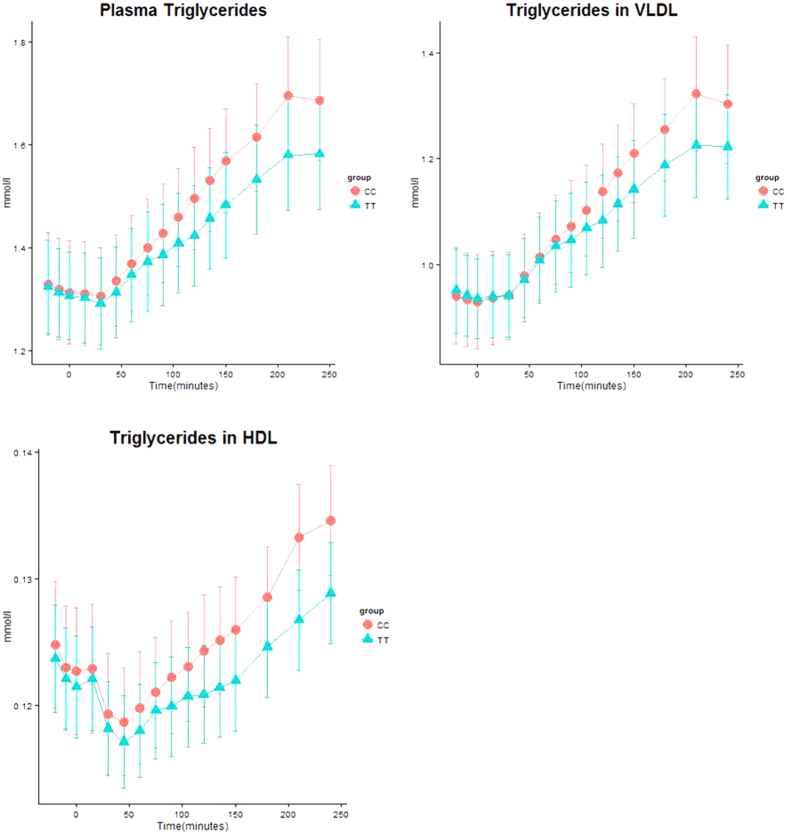
Triglyceride levels during a mixed meal test among TT and CC carriers. Data is expressed as mean ± SEM.

Dynamic response curves of total triglycerides, VLDL-TG and HDL-TG are depicted in Fig. 2.

## Discussion

In this study, we present the effects of the minor T-allele of *TCF7L2* rs7903146, the strongest T2D susceptibility gene variant, on postprandial lipid responses in homozygous carriers. Using a metabolomics platform allowing deep characterization of metabolite profiles, we demonstrate that TT carriers have significantly altered postprandial lipid response in addition to the previously established effect on glucose metabolism. The rate of change (concentration/min) of several subclasses of lipid particles are lower in high risk TT carriers during a meal test, with a strong effect on triglyceride levels suggesting a genotype-mediated effect on postprandial triglyceride regulation.

Several studies have suggested an association between *TCF7L2* rs7903146 and dyslipidemia^14^,^15^,^16^,^17^, and recently a meta-analysis including 24 studies with 52,575 individuals demonstrated that the minor T-allele was associated with lower triglyceride levels in individuals with T2D^11^. Despite a high number of study participants, the meta-analysis was unable to demonstrate an association between the T-allele and triglyceride levels in nondiabetic carriers.

In the present study, we report that normoglycemic homozygous T-allele carriers have marked alterations in their postprandial lipid response following a meal test. We demonstrate that T-allele carriers have lower postprandial triglyceride-rich lipoproteins, with levels of VLDL-TG (x-small, small and medium), HDL-TG (small) and total triglycerides in plasma reaching statistical significance. Our results are diverging from two previous studies. Perez-Martinez *et al*. found that young male carriers of the rs7903146 T-allele have lower HDL and Apo A1 response to a saturated fatty acid-rich meal, while healthy elderly persons have higher levels of plasma cholesterol and Apo B with accumulation of Apo B, cholesterol and triglycerides in small-triglyceride rich lipoproteins^18^. The studies may not be directly comparable since Perez-Martinez *et al*. used a fatty acid rich meal (65% fat, 10% protein and 25% carbohydrate), while this study used a standard breakfast meal (34% fat, 19% protein and 47% carbohydrate). Musso *et al*. found a higher incremental area under curve of triglycerides in healthy controls, suggesting altered postprandial triglyceride response in carriers of the T-allele. Similarly, Musso *et al*. used a high fat meal (the amount of fat was based on body surface area with 78.3 g fat, 55.6% saturated fatty acids, 29.6% monounsaturated fatty acids, 4.2% polyunsaturated fatty acids, 0.5 g cholesterol per m^2^)^19^. Subsequently, dissimilarities in postprandial triglyceride response between our and previous studies may be related to the study design and choice of meal test. However, it was recently demonstrated that the fat content in a meal test is not directly associated with postprandial triglyceride response^20^.

We found minor differences between CC and TT carriers in fasting lipid levels (Ratio of LA/FA, and levels of L-VLDL-L, -C and –CE) indicating that the majority of the genotype-mediated effect on lipids arise postprandially. The concentration of chylomicrons (measured as XXL-VLDL) did not show substantial difference between the groups, suggesting that the lower triglyceride levels among TT carriers are not caused by decreased intestinal uptake and transport of triglycerides from the intestine to the liver. Rather, the lower plasma levels of triglycerides in TT carriers may be liver-mediated and possibly influenced by uptake, synthesis or degradation of triglyceride-rich lipid particles.

A recent study by Norton *et al*. demonstrated that TCF7L2 in a time-dependent manner regulates the expression of 406 genes in hepatocytes^10^. Strong proximal TCF7L2 binding indicated direct regulation of 149 genes^10^. Silencing of *TCF7L2* resulted in altered expression of multiple genes involved in lipid uptake, synthesis, storage and transport, indicating that reduced gene expression leads to increased synthesis of cholesterol and triglycerides. Animal studies have suggested that *TCF7L2* may regulate lipid levels through an insulin independent manner. In mice, with impaired Wnt signaling due to a LRP6 mutation, TCF7L2 expression is reduced, and the animals exhibit increased de novo lipogenesis and cholesterol synthesis leading to high plasma triglyceride levels^21^. Additionally, Boj *et al*. reported that newborn liver-specific *TCF7L2* knockout mice have altered hepatic lipid metabolism with significantly lower serum levels of triglycerides and fatty acids^6^. In summary, animal studies report an association between *TCF7L2* and hepatic lipid regulation, although, the directionality of the effect is not clear. The expression and splicing of *TCF7L2* is highly tissue-specific, and altered expression and alternative splicing of mRNA transcripts may influence the function of TCF7L2. This could provide an explanation for the diverging results on triglyceride levels in knockout models^22^,^23^. To test the hypothesis that TCF7L2 influence hepatic lipid regulation in an insulin independent manner we tested our mixed models with and without adjustments for insulin levels. We found no changes in significance levels among the reported lipid fractions, which may to some extent, support the findings from animal studies that the role of TCF7L2 works in hepatic lipid regulation is independent of insulin.

Triglycerides are transported together with cholesterol in triglyceride-rich lipoproteins, including VLDLs, chylomicrons and corresponding remnants. Triglycerides have been associated with development of cardiovascular disease; however, the causal relationship between triglycerides and development of atherosclerosis is still elusive^24^,^25^,^26^. It has been suggested, that triglycerides are not causing atherosclerosis but are rather a marker of the cholesterol-content in triglyceride-rich lipoproteins^27^,^28^. Nonetheless, triglyceride-rich lipoproteins are able to penetrate the arterial intima causing low-grade inflammation due to triglyceride hydrolysis and formation of macrophage foam cells, leading to increased risk of atherosclerotic cardiovascular disease^26^.

In this study, we report postprandial changes in lipids trying to mimic the daily physiologic excursions in lipid levels among *TCF7L2* variant carriers. Indeed, the postprandial state may have substantial impact on risk of atherosclerosis since many hours daily are spend in the postprandial state. In line with this, previous studies have suggested that non-fasting triglyceride levels may be better predictors of cardiovascular disease risk than fasting levels of triglycerides^27^,^29^,^30^. This could indicate that non-diabetic carriers of the T-allele have a decreased risk of developing atherosclerosis and cardiovascular disease.

Yet, previous studies have found a positive association between the *TCF7L2* rs790346 T- allele and development of coronary artery disease (CAD) in diabetic patients^17^. Causality, however, is difficult to establish since T2D per se amplifies the risk of atherosclerosis and CVD^31^,^32^. Therefore, the association between the T-allele and CAD may not be accurate and further studies are needed to assess this association.

In conclusion, our study demonstrates the complexity in determining the exact role of TCF7L2 in human metabolism, since changes in expression may cause different or even diverging effects across tissues. Nonetheless, our detailed characterization of dynamic metabolite changes during a meal test, suggest that T-allele carriers have altered lipid metabolism, possibly mediating a protective effect against elevated triglycerides and triglyceride-rich particles, which may translate in to a decrease in risk of developing atherosclerosis.

## Methods

Detailed description of the study design, experimental procedures and study participants have been published previously^33^. All participants were recruited in 2007 based on their *TCF7L2* genotype from the population-based Inter99 study conducted at the Research Centre for Prevention and Health, Glostrup, Denmark^34^.

### Study subjects

Sixty-two men were examined, 31 homozygous for the high risk *TCF7L2* rs7903146 T allele (cases) and 31 age- and BMI-matched carriers of the low-risk *TCF7L2* rs7903146 C allele (controls). All participants were glucose tolerant and had no concurrent medical conditions. All participants gave details on medication and prescriptions. Three individuals were treated for hypercholesterolemia, and four for hypertension.

### Meal test

All participants consumed a test meal at 8:00 in the morning after approximately 12 hours of fasting. The meal was consumed within 15 minutes. The mixed meal consisted of 50 g white bread, 50 g black bread, 10 g butter, 40 g cheese, 20 g sugar-free jam and 200 ml milk (34% fat, 47% carbohydrate, 19% protein), comprising a total of 2,370 kJ (566 kcal). The mixed meal test was chosen to mimic a standard breakfast. Arterialised venous blood was drawn at 20, 10 and 0 min before and at 15, 30, 45, 60, 75, 90, 105, 120, 135, 150, 180, 210 and 240 min after ingestion of the meal.

### Blood samples

All blood samples were drawn prior to, during and after a meal test. Samples included EDTA plasma and were kept in a freezer at −80 °C.

### Metabolite profiling

Samples were analysed for levels of metabolites at the 16 time points including a full set of molecular measures quantified from native plasma by targeted NMR (nuclear magnetic resonance) profiling. The high-throughput NMR platform (Brainshake Ltd, Helsinki, Finland) has previously been used in various epidemiological and genetic studies and details of the experimental protocols, including sample preparation and spectroscopy, have been described elsewhere^35^,^36^.

The NMR platform quantifies the number and size of lipoproteins, each expressed as a lipoprotein particle concentration (mmol/L, mol/L or g/L), or as an average particle size (nanometers). The concentrations of lipoproteins of different sizes were calculated from the measured amplitudes of their spectroscopically distinct lipid methyl-group NMR signals. Weighted-average lipoprotein particle sizes were derived from the sum of the diameter of each subclass multiplied by its relative mass percentage based on the amplitude of its methyl NMR signal^35^,^36^.

Metabolites assessed included 141 different lipid particles: lipoprotein particles and subclasses (chylomicrons, very large density lipoproteins (VLDL). intermediate density lipoproteins (IDL), large density lipoproteins (LDL), high density lipoproteins (HDL) particles), cholesterols (Total, VLDL, LDL, HDL, Free cholesterol and esterified cholesterol), glycerides and phospholipids (triglycerides in VLDL, LDL and HDL, diglycerides, phosphoglycerides, phosphatidylcholine and sphingomyelins), apolipoproteins (ApoA1, ApoB, ApoB/ApoA1), fatty acids (total fatty acids, degree of saturation, docosahexaenoic acid, linoleic acid, omega-3-fatty acids, omega-6-fatty acids, polyunsaturated fatty acids, monounsaturated fatty acids, saturated fatty acids).

### Statistical analysis

Statistical analyses were performed using R, version 3.1.3 and 3.3.0 (http://www.r-project.org). Data is expressed as medians (range: minimum to maximum) or means ± standard deviation. Demographic variables were compared by either Fishers exact test (physical exercise) or student´s t test. Glucose, insulin and ALAT levels were log transformed prior to analysis. Insulin resistance was evaluated by the homeostatic model assessment of insulin resistance (HOMA- IR) in the fasting state (HOMA IR = (glucose (mmol/L) × insulin (pmol/L))/22.5). All fasting metabolite levels were compared at 0 minutes.

Metabolite levels during the meal test were compared using linear mixed models. All metabolites were pareto-scaled (divided by the square root of the standard deviation) prior to analysis. Linear mixed models were chosen to assess the effect of genotype on the course of the curve (time-genotype interaction) during the meal test from 0 to 240 min. Gaussian spatial correlation was used to model the covariance allowing metabolite measurements that are temporally close together to be correlated to a higher degree than measurements further apart. Model assumptions were checked visually. False-discovery rates of 5% were applied to account for multiplicity. Similarly, the p-values were Bonferroni-corrected by multiplying by the number of comparisons (number of time points and metabolites, n = 2304). The mixed linear models were compared by an anova to mixed linear models adjusted for insulin levels in order to detect if significant findings were insulin-dependent.

After conducting the linear mixed models, we tested if there were any clustering of metabolites in relation to relative changes in concentrations of metabolites during the meal test. To do so, we visualized the relative changes of each metabolite and compared them by group. Absolute metabolite concentrations per time point were calculated by taking the log10 of the ratio to the mean (over all time points) for each metabolite and for each study participant. From those log-transformed ratios, the relative change to the preceding time point was calculated for all time points except the first (e.g. log10-ratio at 210 minutes - log10–ratio at 180 minutes). A Wilcoxon-Mann-Whitney test was performed to compare the relative changes of all metabolites by comparing the distributions between the two genotypes (CC and TT).

## Additional Information

**How to cite this article:** Engelbrechtsen, L. *et al*. Homozygous carriers of the *TCF7L2* rs7903146 T-allele show altered postprandial response in triglycerides and triglyceride-rich lipoproteins. *Sci. Rep.* **7**, 43128; doi: 10.1038/srep43128 (2017).

**Publisher's note:** Springer Nature remains neutral with regard to jurisdictional claims in published maps and institutional affiliations.

## Supplementary Material

Supplementary Table

## Figures and Tables

**Table 1:** Baseline characteristics of study participants.

Variable	TT-carriers	CC-carriers	p-value
Age (years)	53.3 ± 6.3	53.6 ± 6.9	0.7
BMI (kg/m^2^)	25.7 ± 2.5	26.3 ± 3.1	0.1
Waist-hip ratio	0.9 ± 0.1	0.9 ± 0.04	1.0
HOMA IR	9.6 ± 6.2	9.9 ± 6.1	1.0
Glucose (mmol/l)	5.7 ± 0.44	5.53 ± 0.37	0.1
Insulin (mmol/l)	36.4 ± 18.3	43.2 ± 25.5	0.2
ALAT (mmol/l)	3.18 ± 0.49	3.26 ± 0.42	0.5
Exercise (self-reported, excluding work) Inactive or <2 h/week (very light exercise)	1 (3%)	0	1.0
2–4 h/week (light exercise)	17 (55%)	17 (55%)	1.0
>4 h/week of moderate exercise or 2–4 h/week of moderate exercise	12 (39%)	14 (45%)	0.80
>4 h/week of vigorous exercise	1 (3%)	0	1.0

Data expressed as means ± SD or n(%). Concentrations were measured at fasting. Demographic description of study participants have been published previously^13^. Glucose, insulin and ALAT were log transformed prior to analysis. Statistical analyses used were students T test and Fishers exact test.

**Table 2:** Metabolite responses to a meal test in TT and CC carriers of the rs7903146 *TCF7L2.*

Metabolites	TT-carriers		CC-carriers		P	FDR	Bonferroni
	Fasting	240 min	Fasting	240 min			
Total Cholesterol	4.7 (3.1–6.3)	4.6 (2.9–6.5)	5.0 (3.0–6.6)	4.9 (3.0–6.9)	0.27	0.42	1
Total TG (mmol/l)	1.2 (0.6–2.5)	1.4 (0.5–3.3)	1.3 (0.5–2.7)	1.4 (0.4–3.2)	0.0002	0.005	0.030*
**VLDLs**							
VLDL-TG (mmol/l)	0.9 (0.3–2.0)	1.0 (0.3–2.9)	0.9 (0.3–2.3)	1.0 (0.2–2.8)	0.0003	0.005	0.05*
XL-VLDL-TG (mmol/l)	0.04 (0.0–0.2)	0.05 (0–0.3)	0.04 (0.0–0.2)	0.05 (0–0.2)	0.009	0.04	1
L-VLDL-TG (mmol/l)	0.2 (0.02–0.5)	0.2 (0.01–0.8)	0.2 (0.01–0.6)	0.2 (0–0.8)	0.001	0.01	0.20
M-VLDL-TG (mmol/l)	0.3 (0.1–0.7)	0.3 (0.1–1.0)	0.3 (0.09–0.8)	0.3 (0.1–1.0)	0.0003	0.005	0.048*
S-VLDL-P (mol/l)	3.5 × 10^−8^ (1.9–5.4 × 10^−8^)	3.6 × 10^−8^ (1.8–6.1 × 10^−8^)	3.6 × 10^−8^ (2.0–6.1 × 10^−8^)	3.7 × 10^−8^ (1.8–6.3 × 10^−8^)	0.0002	0.005	0.031*
S-VLDL-TG (mmol/l)	0.3 (0.1–0.5)	0.3 (0.1–0.5)	0.2 (0.1–0.5)	0.3 (0.09–0.5)	5.98 × 10^−6^	0.0005	0.0009*
XS-VLDL-TG (mmol/l)	0.1 (0.06–0.2)	0.1 (0.05–0.2)	0.1 (0.06–0.2)	0.1 (0.05–0.2)	0.0003	0.005	0.044*
**LDLs**							
L-LDL-TG (mmol/l)	0.09 (0.05–0.12)	0.08 (0.04–0.1)	0.09 (0.05–0.15)	0.08 (0.05–0.2)	0.52	0.67	1
M-LDL-TG (mmol/l)	0.04 (0.01–0.05)	0.03 (0.009–0.6)	0.04 (0.02–0.06)	0.03 (0.01–0.07)	0.34	0.50	1
S-LDL-TG (mmol/l)	0.02 (0.007–0.04)	0.02 (0.004–0.04)	0.02 (0.01–0.04)	0.02 (0.007–0.04)	0.007	0.034	1
**HDLs**							
HDL-TG (mmol/l)	0.12 (0.08–0.16)	0.1 (0.08–0.2)	0.12 (0.08–0.19)	0.1 (0.08–0.2)	0.0002	0.005	0.037*
XL-HDL-TG (mmol/l)	0.017 (0.01–0.03)	0.02 (0.01–0.03)	0.017 (0.01–0.03)	0.02 (0–0.03)	0.07	0.15	1
L-HDL-TG (mmol/l)	0.02 (0.01–0.04)	0.02 (0.01–0.04)	0.03 (0.02–0.05)	0.03 (0.01–0.05)	0.43	0.59	1
M-HDL-TG (mmol/l)	0.04 (0.02–0.05)	0.03 (0.01–0.06)	0.04 (0.02–0.07)	0.03 (0.01–0.07)	0.007	0.034	1
S-HDL-TG (mmol/l)	0.04 (0.03–0.06)	0.04 (0.02–0.07)	0.04 (0.02–0.08)	0.04 (0.02–0.08)	1.78 × 10^−7^	2.83 × 10^−5^	2.83 × 10^−5^*
**Fatty acids**							
Total fatty acids	11.5 (7.7–15.5)	11.7 (7.1–17.6)	12.1 (8.0–15.9)	12.2 (7.7–16.4)	0.056	0.15	1
MUFA	2.9 (1.6–4.5)	2.9 (1.3–5.4)	2.9 (1.8–5.4)	3.0 (1.8–5.8)	0.003	0.016	0.445
MUFA/FA (%)	25.3 (20.4–31.7)	25.1 (18–31.8)	25.4 (21.1–34.3)	25.5 (16–35.1)	3.72 × 10^−5^	0.002	0.006*
PUFA	4.5 (3.1–5.7)	4.4 (2.8–5.7)	4.5 (3.1–5.7)	4.5 (2.9–6.2)	0.25	0.40	1

Data is expressed as median (range) at fasting and at 240 min after ingestion of a meal. Mixed models were used for calculation of p-values, comparing the postprandial metabolite responses from 0 min to 240 min. P-values are crude values. FDR states p-values adjusted for a false discovery rate of 5%, and Bonferroni is the p-values corrected for multiple testing by Bonferroni correction. S = small size, M = medium size, L = large size, XL = extra large size. TG = triglycerides. MUFA = Monounsaturated fatty acids. PUFA = Polyunsaturated fatty acids.
