# Supplementary material for: Homozygous carriers of the TCF7L2 rs7903146 T-allele show altered postprandial response in triglycerides and triglyceride-rich lipoproteins
Source: Sci Rep. 2017 Feb 21;7:43128. doi: 10.1038/srep43128 (PMC5318936; doi:10.1038/srep43128)
Supplement: Supplementary Table [file srep43128-s1.pdf]

SUPPLEMENTARY

Homozygous carriers of the *TCF7L2* rs7903146 T-allele show altered postprandial response in triglycerides and triglyceride-rich lipoproteins

Engelbrechtsen L[1,2], Hansen TH[1], Mahendran Y[1,2], Pyl P[1], Andersson E.[1] , A. Jonsson[1], A. Gjesing[1], A. Linneberg[3,4,5], T. Jørgensen[3,6,7], T. Hansen[1] and H. Vestergaard[1,8]

Table 1: The effect of genotype and time on metabolite changes during a meal test

Metabolite	Estimate	2.5%	97.5%","	P	FDR	Bonferroni
S.HDL.TG	-4.90E-10	-9.27E-10	-1.91E-10	1.78E-07	2.83E-05	2.83E-05
S.VLDL.TG	-5.08E-09	-1.04E-08	-1.64E-09	5.98E-06	0.000475	0.00095
MUFA.FA	-7.68E-08	-1.67E-07	-2.12E-08	3.72E-05	0.001971	0.005914
M.VLDL.P	-5.52E-16	-1.32E-15	-1.13E-16	0.000344	0.004847	0.054685
M.VLDL.L	-1.75E-08	-4.23E-08	-3.50E-09	0.000396	0.004847	0.063016
M.VLDL.PL	-3.14E-09	-7.55E-09	-6.34E-10	0.000371	0.004847	0.059031
M.VLDL.FC	-2.32E-09	-5.57E-09	-4.70E-10	0.000365	0.004847	0.058007
M.VLDL.TG	-1.22E-08	-2.90E-08	-2.55E-09	0.000302	0.004847	0.047956
S.VLDL.P	-2.13E-16	-4.95E-16	-4.78E-17	0.000195	0.004847	0.03097
XS.VLDL.TG	-7.59E-10	-1.80E-09	-1.61E-10	0.000275	0.004847	0.043789
Plasma.TG	-3.07E-08	-7.15E-08	-6.92E-09	0.000191	0.004847	0.030437
VLDL.TG	-3.42E-08	-8.16E-08	-7.08E-09	0.000322	0.004847	0.051225
HDL.TG	-8.69E-10	-2.04E-09	-1.90E-10	0.000235	0.004847	0.037428
S.VLDL.L	-3.28E-09	-8.04E-09	-6.24E-10	0.000513	0.005828	0.081592
ApoB.ApoA1	-9.35E-10	-2.33E-09	-1.65E-10	0.000718	0.007606	0.114086
M.VLDL.C	-3.16E-09	-7.93E-09	-5.48E-10	0.000784	0.007788	0.124605
L.HDL.FC	8.12E-10	1.35E-10	2.06E-09	0.000942	0.008808	0.149741
L.VLDL.P	-3.62E-16	-9.42E-16	-5.41E-17	0.001397	0.010279	0.222088
L.VLDL.L	-2.05E-08	-5.37E-08	-2.98E-09	0.00153	0.010279	0.243236
L.VLDL.PL	-3.90E-09	-1.02E-08	-5.65E-10	0.001552	0.010279	0.246702
L.VLDL.CE	-1.45E-09	-3.75E-09	-2.23E-10	0.001257	0.010279	0.199888
L.VLDL.TG	-1.34E-08	-3.46E-08	-2.04E-09	0.001304	0.010279	0.207382
S.VLDL.PL	-5.43E-10	-1.42E-09	-8.00E-11	0.001468	0.010279	0.233364
S.VLDL.FC	-3.74E-10	-9.78E-10	-5.49E-11	0.001489	0.010279	0.23678
VLDL.D	-6.85E-09	-1.81E-08	-9.65E-10	0.001706	0.010853	0.271329
L.HDL.C	3.15E-09	4.24E-10	8.39E-09	0.001958	0.011973	0.311304
MUFA	-2.52E-08	-6.92E-08	-2.99E-09	0.002802	0.016499	0.445468
L.VLDL.C	-3.39E-09	-9.39E-09	-3.78E-10	0.003239	0.017166	0.514967
M.VLDL.CE	-1.11E-09	-3.08E-09	-1.26E-10	0.003144	0.017166	0.499874
L.HDL.CE	2.29E-09	2.63E-10	6.33E-09	0.003034	0.017166	0.482342
HDL2.C	2.91E-09	2.50E-10	8.48E-09	0.00555	0.028466	0.88244
L.VLDL.FC	-2.06E-09	-6.08E-09	-1.63E-10	0.0064	0.031799	1

S.LDL.TG	-2.37E-10	-7.08E-10	-1.76E-11	0.007071	0.034072	1
L.HDL.L	4.81E-09	3.43E-10	1.45E-08	0.007501	0.034076	1
M.HDL.TG	-5.40E-10	-1.62E-09	-3.91E-11	0.007343	0.034076	1
XL.VLDL.TG	-4.38E-09	-1.34E-08	-2.72E-10	0.009056	0.037984	1
XL.HDL.PL	7.19E-10	4.48E-11	2.20E-09	0.009001	0.037984	1
L.HDL.P	7.18E-15	4.45E-16	2.20E-14	0.009078	0.037984	1
XS.VLDL.CE	3.36E-10	1.74E-11	1.05E-09	0.011141	0.045422	1
XL.VLDL.P	-5.82E-17	-1.88E-16	-2.39E-18	0.013985	0.055591	1
XL.VLDL.L	-5.50E-09	-1.79E-08	-2.15E-10	0.014574	0.056519	1
XL.VLDL.CE	-3.81E-10	-1.26E-09	-1.26E-11	0.016573	0.062742	1
L.HDL.PL	2.08E-09	4.38E-11	7.14E-09	0.021838	0.078916	1
VLDL.C	-1.94E-09	-6.75E-09	-3.52E-11	0.023507	0.083056	1
XL.VLDL.C	-6.98E-10	-2.49E-09	-8.60E-12	0.027473	0.091766	1
S.LDL.FC	6.67E-11	8.03E-13	2.38E-10	0.027703	0.091766	1
HDL.C	1.90E-09	2.34E-11	6.80E-09	0.02752	0.091766	1
XL.VLDL.PL	-7.71E-10	-2.77E-09	-8.51E-12	0.028524	0.092558	1
L.LDL.FC	4.59E-10	2.02E-12	1.72E-09	0.035788	0.111449	1
XL.HDL.P	5.95E-16	2.58E-18	2.23E-15	0.035894	0.111449	1
XL.HDL.L	6.44E-10	2.56E-12	2.42E-09	0.036449	0.111449	1
XXL.VLDL.FC	-1.31E-10	-4.99E-10	-2.85E-13	0.039786	0.117148	1
IDL.FC	4.75E-10	1.15E-12	1.81E-09	0.039273	0.117148	1
XL.VLDL.FC	-3.26E-10	-1.26E-09	-3.35E-13	0.042876	0.12395	1
FALen	-1.57E-09	-6.14E-09	-8.85E-13	0.044688	0.126881	1
XS.VLDL.C	3.64E-10	2.62E-14	1.44E-09	0.048074	0.134101	1
TotFA	-1.95E-08	-7.99E-08	-1.35E-11	0.056182	0.153879	1
XXL.VLDL.L	-1.24E-09	-5.26E-09	-4.33E-12	0.064226	0.157376	1
XXL.VLDL.PL	-1.57E-10	-6.59E-10	-3.71E-13	0.061602	0.157376	1
XXL.VLDL.C	-1.48E-10	-6.29E-10	-5.25E-13	0.064336	0.157376	1
XXL.VLDL.TG	-9.57E-10	-4.05E-09	-3.15E-12	0.063793	0.157376	1
M.LDL.FC	9.72E-11	2.81E-13	4.10E-10	0.062885	0.157376	1
XXL.VLDL.P	-5.83E-18	-2.48E-17	-2.46E-20	0.065722	0.157744	1
XL.HDL.TG	-9.70E-11	-4.15E-10	-4.48E-13	0.066471	0.157744	1
S.LDL.C	4.71E-10	3.27E-12	2.05E-09	0.070402	0.164597	1
LA	-2.36E-09	-1.03E-08	-1.80E-11	0.071429	0.164597	1
PC	-1.58E-09	-7.26E-09	-3.23E-11	0.086353	0.196145	1
IDL.C	6.73E-10	2.10E-11	3.19E-09	0.095736	0.214394	1
ApoB	-5.15E-10	-2.48E-09	-1.92E-11	0.100397	0.221711	1
XXL.VLDL.CE	-4.14E-11	-2.05E-10	-2.10E-12	0.109737	0.238507	1
L.LDL.C	9.24E-10	5.33E-11	4.64E-09	0.114003	0.238507	1
S.LDL.CE	3.97E-10	2.23E-11	1.99E-09	0.113068	0.238507	1
LDL.C	1.90E-09	1.07E-10	9.51E-09	0.113119	0.238507	1
FAw6.FA	1.44E-08	9.54E-10	7.34E-08	0.119014	0.245757	1
XL.HDL.FC	8.55E-11	6.45E-12	4.42E-10	0.12412	0.253013	1

IDL.PL	2.84E-10	2.79E-11	1.52E-09	0.135601	0.266713	1
EstC	1.71E-09	1.69E-10	9.17E-09	0.135783	0.266713	1
PUFA.FA	1.48E-08	1.46E-09	7.93E-08	0.135873	0.266713	1
IDL.CE	3.16E-10	3.71E-11	1.74E-09	0.144287	0.279775	1
M.LDL.C	5.03E-10	7.61E-11	2.87E-09	0.158172	0.301592	1
TotCho	-1.18E-09	-6.77E-09	-1.82E-10	0.159332	0.301592	1
L.LDL.CE	4.95E-10	9.86E-11	2.96E-09	0.175312	0.321913	1
XL.HDL.C	2.85E-10	5.70E-11	1.71E-09	0.175593	0.321913	1
FAw3	-2.70E-10	-1.62E-09	-5.45E-11	0.176141	0.321913	1
XL.HDL.CE	2.34E-10	5.90E-11	1.46E-09	0.192138	0.347159	1
L.LDL.L	8.05E-10	2.35E-10	5.19E-09	0.203015	0.36269	1
DHA	-1.10E-10	-7.23E-10	-3.43E-11	0.20819	0.367803	1
IDL.TG	-1.35E-10	-8.91E-10	-4.41E-11	0.212523	0.371331	1
M.LDL.CE	3.95E-10	1.35E-10	2.64E-09	0.216117	0.373507	1
IDL.P	5.39E-17	2.14E-17	3.73E-16	0.229262	0.378128	1
IDL.L	5.72E-10	2.15E-10	3.91E-09	0.224279	0.378128	1
S.LDL.L	2.03E-10	7.66E-11	1.39E-09	0.224547	0.378128	1
TotPG	-8.77E-10	-6.02E-09	-3.36E-10	0.226039	0.378128	1
SFA.FA	7.80E-09	3.15E-09	5.42E-08	0.230682	0.378128	1
PUFA	-1.32E-09	-9.63E-09	-6.49E-10	0.249188	0.404295	1
LDL.TG	-2.33E-10	-1.71E-09	-1.18E-10	0.252084	0.404862	1
ApoA1	2.00E-10	1.11E-10	1.51E-09	0.260867	0.414779	1
Plasma.C	1.17E-09	6.87E-10	8.93E-09	0.267411	0.416846	1
TG.PG	-2.78E-09	-2.13E-08	-1.63E-09	0.266706	0.416846	1
XS.VLDL.FC	6.88E-11	4.74E-11	5.51E-10	0.284164	0.435936	1
L.LDL.P	7.99E-17	5.55E-17	6.42E-16	0.28514	0.435936	1
FAw6	-1.02E-09	-8.24E-09	-7.30E-10	0.288746	0.437244	1
M.LDL.L	3.14E-10	2.51E-10	2.63E-09	0.30088	0.45132	1
L.LDL.PL	9.47E-11	8.06E-11	8.09E-10	0.307995	0.457675	1
S.VLDL.C	-6.68E-11	-6.14E-10	-7.10E-11	0.334632	0.492653	1
M.LDL.TG	-6.83E-11	-6.45E-10	-7.86E-11	0.344419	0.502409	1
S.LDL.P	3.92E-17	5.65E-17	4.02E-16	0.373027	0.534337	1
M.LDL.P	4.48E-17	7.32E-17	4.82E-16	0.389496	0.552945	1
SFA	-2.47E-09	-2.78E-08	-4.55E-09	0.405734	0.5709	1
L.HDL.TG	-9.99E-12	-1.22E-10	-2.24E-11	0.432634	0.594434	1
Remnant.C	-2.26E-10	-2.78E-09	-5.11E-10	0.433675	0.594434	1
FAw3.FA	7.72E-10	1.71E-09	9.40E-09	0.430875	0.594434	1
DHA.FA	2.86E-10	1.04E-09	4.37E-09	0.50048	0.663136	1
L.LDL.TG	-3.80E-11	-6.17E-10	-1.56E-10	0.517263	0.670335	1
S.HDL.PL	-5.40E-11	-8.79E-10	-2.24E-10	0.518561	0.670335	1
LA.FA	1.66E-09	6.85E-09	2.69E-08	0.518323	0.670335	1
XS.VLDL.PL	3.06E-11	1.62E-10	5.67E-10	0.55253	0.708486	1
XS.VLDL.L	4.68E-11	2.92E-10	9.48E-10	0.57531	0.731756	1

UnSat	3.98E-11	2.57E-10	8.21E-10	0.579882	0.731756	1
S.VLDL.CE	1.35E-11	1.08E-10	3.15E-10	0.60903	0.762486	1
M.HDL.CE	5.48E-11	5.11E-10	1.40E-09	0.628658	0.780911	1
M.HDL.C	6.57E-11	7.19E-10	1.85E-09	0.649195	0.790779	1
SM	-9.44E-11	-2.87E-09	-1.16E-09	0.663927	0.799075	1
CLA.FA	2.08E-09	2.66E-08	6.46E-08	0.668409	0.799075	1
S.HDL.P	-1.81E-16	-7.54E-15	-3.59E-15	0.71959	0.831301	1
S.HDL.C	4.32E-11	8.38E-10	1.77E-09	0.716777	0.831301	1
S.HDL.CE	4.29E-11	8.60E-10	1.80E-09	0.720438	0.831301	1
DAG	6.20E-11	3.20E-09	5.23E-09	0.810741	0.901453	1
S.HDL.FC	2.23E-12	1.26E-10	2.01E-10	0.817787	0.902973	1
S.HDL.L	-1.44E-11	-1.56E-09	-1.02E-09	0.834897	0.909237	1
CLA	5.91E-11	3.88E-09	6.03E-09	0.8296	0.909237	1
M.HDL.FC	2.25E-12	2.85E-10	3.95E-10	0.872802	0.944052	1
M.HDL.PL	8.54E-12	1.28E-09	1.74E-09	0.882504	0.948095	1
FreeC	-6.14E-12	-1.81E-09	-1.42E-09	0.903574	0.953111	1
DAG.TG	1.36E-11	2.62E-09	3.43E-09	0.895047	0.953111	1
XS.VLDL.P	6.99E-20	3.80E-17	4.47E-17	0.935702	0.959595	1
M.LDL.PL	-2.20E-13	-1.99E-10	-1.73E-10	0.946215	0.959595	1
S.LDL.PL	-1.25E-13	-9.58E-11	-8.24E-11	0.941349	0.959595	1
M.HDL.L	5.95E-12	2.56E-09	3.08E-09	0.928165	0.959595	1
HDL3.C	4.46E-13	3.70E-10	4.23E-10	0.947525	0.959595	1
M.HDL.P	1.17E-19	6.65E-15	6.76E-15	0.993468	0.993468	1

The estimates represent the interaction between time and genotype (concentration/ minute).
